# 

**Published:** 1893-12-02

**Authors:** 


					HOSPITAL. - December 2nd, 1893.
PRICE TWOPENCE. ^ WW WITH EXTRA nursing supplement.
. 375, Vol. XV.?Deo. 2nd, 1893. <HT^ " In* IfiP >??""??
-gistered at the General Post Office as a Newepaper for ''lL JMC . / /.-/? Monthly Parts, 6<L ; post free, 3d.
transmission in the United Kingdom and Abroad.] ( / 7 Ditto per Annum, 8s., post free.
No. 375. Vol. XY. Saturday,
December. 2nd, 1893.
[Twopence.

				

